# Circular RNA Circ_0038467 promotes the maturation of miRNA-203 to increase lipopolysaccharide-induced apoptosis of chondrocytes

**DOI:** 10.1515/med-2022-0557

**Published:** 2023-06-05

**Authors:** Zhongkun Gou, Quanling Wu, Changqing Jiang, Wei Dong

**Affiliations:** Department of Bone and Joint Surgery, Shenzhen Baoan Shiyan People’s Hospital, Shenzhen, Guangdong Province, 518108, PR China; Department of Orthopedics, Shenzhen Baoan Shiyan People’s Hospital, Shenzhen, Guangdong Province, 518108, PR China; Department of Sports Medicine, Peking University Shenzhen Hospital, Shenzhen City, Guangdong Province, 518036, PR China

**Keywords:** Circ_0038467, osteoarthritis, miR-203, chondrocytes, maturation

## Abstract

Circ_0038467 and miR-203 exert important functions in lipopolysaccharide (LPS)-induced inflammation, which contributes to osteoarthritis (OA). Our preliminary deep sequencing analysis revealed altered expression of Circ_0038467 and miR-203 in OA and a close correlation between them. This study was therefore to explore crosstalk between them in OA. The expression of Circ_0038467, mature miR-203, and miR-203 precursor in OA patients and controls was determined using RT-qPCR. An overexpression assay was performed to explore the role of Circ_0038467 in regulating the expression of mature miR-203 and miR-203 precursor. Cell apoptosis was analyzed by cell apoptosis assay. Circ_0038467 was upregulated in OA and positively correlated with mature miR-203 but not that of miR-203 precursor. In chondrocytes, increased expression levels of both Circ_0038467 and miR-203 were observed after LPS treatment. In chondrocytes, overexpression of Circ_0038467 increased the expression levels of mature miR-203 but not that of miR-203 precursor. Overexpression of Circ_0038467 and miR-203 increased cell apoptosis. Then, the miR-203 inhibitor reversed the effects of overexpression of Circ_0038467 on cell apoptosis. Interestingly, Circ_0038467 was detected in both the cytoplasm and nucleus. Circ_0038467 directly interacted with the precursor miR-203. Therefore, Circ_0038467 is highly expressed in OA and it may promote the production of mature miR-203 to increase apoptosis of chondrocytes induced by LPS.

## 1 Introduction

With aging, there is a loss and degradation of the articular cartilage, leading to the development of osteoarthritis (OA), which is a type of chronic degenerative disease [1]. Among the population older than 60 years, more than 13% of females and 10% of males are suffering from OA [2]. Despite the advances in the diagnosis and treatment of OA, there is still no cure for OA [3]. The currently available treatment approaches mainly focus on symptom relief [4]. For instance, acetaminophen and nonsteroidal anti-inflammatory drugs can be used to relieve chronic pain [4]. Even worse, these treatments may not work on severe OA [3,4]. More effective treatment approaches have been developed in the past few decades. To date, pathological changes of OA, such as the destruction and loss of articular cartilage, still cannot be reversed by any available approaches. To this end, novel therapeutic approaches are needed to improve the quality of patients’ life by stimulating the formation of new cartilage. However, the pathogenesis of OA remains largely unknown, which limits the discovery of novel treatments [5,6].

Molecular factors play important roles in the development and progression of OA [7,8]. Some pathways, such as the Notch pathway, NF-κB pathway, and PCP/JNK-mTORC1-PTHrP cascade, have been demonstrated to be critical players in OA [7,8]. In effect, some molecular factors are promising targets for the targeted therapy of OA [9,10]. However, safer and more effective targets for OA remain to be developed. CircRNAs are closed (covalent bond) RNA transcripts with critical functions in the regulation of gene expression rather than coding proteins [11,12,13]. In OA, CircRNAs not only participate in disease progression but also serve as biomarkers for the diagnosis and prognosis of diseases [11]. It is well known that changes in the accumulation of CircRNAs reflect the initiation and progression of human diseases, including OA [11], suggesting the potential role of CircRNAs as promising targets for targeted OA therapy. Circ_0038467 is a newly identified CircRNA produced from the back-splicing of ubiquinol-cytochrome-c reductase core protein 2 (UQCRC2) gene [14]. The development of OA is closely associated with lipopolysaccharide (LPS)-induced inflammation [12], in which Circ_0038467 plays a critical role [13]. For instance, increased LPS accumulation induces inflammatory responses and altered LPS clearance promotes the progression of OA [13]. Therefore, Circ_0038467 is likely involved in OA. Our deep sequence analysis revealed the altered expression of Circ_0038467 and its inverse correlation with miR-203 (data not shown), which is also a critical player in LPS-induced cell injury [15]. For instance, the knockdown of miR-203 can target MCL-1 to alleviate LPS-induced injury in C28/I2 chondrocytes [15]. Therefore, Circ_0038467 may interact with miR-203 to participate in OA. We, therefore, explored the potential interaction between Circ_0038467 and miR-203 in OA.

Our study reported the involvement of Circ_0038467 in OA and its regulatory role in the maturation of miR-203. Our study is the first to report the regulation of miR-203 maturation by a CircRNA in any physiological and pathological processes. Regulation of the Circ_0038467/miR-203 axis may serve as a novel target to treat OA. However, animal model experiments and clinical trials are still needed to explore the clinical value of this novel axis of Circ_0038467/miR-203 in the treatment of OA.

## 2 Materials and methods

### 2.1 Participants

In this study, we enrolled 60 controls (20 males and 40 females) and 60 OA patients (hip OA, 20 males and 40 females) at Shenzhen Baoan Shiyan People’s Hospital between June 2018 and June 2020. The inclusion criteria were as follows: (1) OA patients diagnosed according to the 1986 classification of OA of the knee in diagnostic criteria of the American Rheumatism Association; (2) Kellgren–Lawrence (KL) grade II–III; and (3) pain of knee joint lasted for at least 1 month and Western Ontario and McMaster Universities Osteoarthritis Index (WOMAC) pain score was above 40 mm. Exclusion criteria were as follows: (a) patients complicated with other arthritis diseases such as rheumatoid arthritis, septic arthritis, osteochondritis dissecans, or spondylarthritis; (b) patients complicated with congestive heart failure, unstable angina, uncontrolled hypertension, stroke, or transient ischemic attack within 6 months; (c) patients complicated with severe renal or hepatic dysfunction; (d) knee OA with two‐side joints; (e) history of hip joint surgery; (f) received NSAIDs or high‐dose acetaminophen treatment within 1 month; (g) received an articular injection of glucocorticoid or hyaluronic acid (HA) sodium within 3 months; and (h) pregnancy or lactation. Healthy controls were enrolled at the physiological health center. The physiological functions of all organs of the healthy controls were within the normal range. The age range of both PA patients and healthy controls was 56–70 years, with a median age of 63 years. Clinical data of both OA patients and healthy controls are presented in Table 1. No significant differences in age, gender, obesity, and habits of smoking and drinking were found between the two groups.

**Table 1:** Clinical data of both OA patients and healthy controls

	OA (*I* = 60)	Control (*n* = 60)
**Gender**		
Male	20 (33.33%)	20 (33.33%)
Female	40 (66.67%)	40 (66.67%)
**Age (years, mean ± SD)**	63.72 ± 7.11	63.41 ± 8.11
56–63	21 (35%)	26 (43.33%)
64–70	39 (65%)	34 (56.67%)
Body mass index (kg/m^2^)	23.1 ± 2.23	22.2 ± 2.04
Disease duration (months)	72.34 ± 14. 67	NA
**KL stage**		
III	26 (43.33%)	NA
IV	34 (56.67%)	NA
Strength of the knee extensor (N/kg)	2.6 ± 0.77	2.8 ± 0.75
Strength of the hip abductor (N/kg)	0.8 ± 0.34	0.94 ± 0.31


**Ethical approval and consent to participate:** Informed consent was obtained from all individual participants included in the study. All procedures were approved by the Shenzhen Baoan Shiyan People’s Hospital Ethics Committee. Procedures operated in this research were completed in keeping with the standards set out in the Announcement of Helsinki and laboratory guidelines of research in China.

### 2.2 Synovial fluid, treatment, and chondrocytes derived from OA patients

Under local anesthesia, a syringe was used to extract the synovial fluid (1.5–2.0 ml) from the affected hip of OA patients. To serve as the control, a similar amount of the synovial fluid was also extracted from the hip of healthy controls. All patients were treated with NSAIDs. Different patients received different NSAIDs and dosages varied across patients. NSAIDs exert their anti-inflammatory and analgesic effects to alleviate OA by inhibiting the prostaglandin-generating enzyme, cyclooxygenase. At 1 month after treatment, symptoms were improved in all cases. At this time point, the synovial fluid was also extracted from OA patients. The synovial fluid samples were kept in liquid nitrogen storage prior to the subsequent assays.

OA-affected chondrocytes (402OA-05A; Sigma-Aldrich) were also used in this study. Chondrocyte growth medium, purchased from PromoCell (Heidelberg, Germany), was used to cultivate cells in an incubator at 37°C with 5% CO_2_ and 95% humidity. To study the effects of LPS treatment on gene expression, chondrocytes were cultivated in a medium containing 0, 2, 4, 8, and 10 ng/ml LPS (Cat # L2630-10MG; Sigma-Aldrich) for 48 h prior to the subsequent assays. Three biological replicates were set for each experiment.

### 2.3 Cell transfection

The expression vector of Circ_0038467 [13] was established using pcDNA3.1(+) CircRNA Mini Vector (Addgene) as the backbone. Mimics of miR-203 and miRNA precursor as well as miR-203 inhibitor (5′-GUGAAAUGUUUAGGACCACUAG-3′) and negative control (NC, Cat # AM17010, Thermo Fisher) were used. Lipofectamine 2000 (Invitrogen) was used to transfect 1 µg of the Circ_0038467 expression vector, 40 nM miR-203 inhibitor, or 40 nM miR-203 mimic into 10^7^ chondrocytes. Control (untransfected cells, C) and NC (NC miRNA-, NC inhibitor- or empty vector-transfected cells) experiments were also included. Cells were incubated with the transfection mixture for 6 h, followed by washing with a fresh medium to reduce cytotoxic.

### 2.4 RNA isolation and genomic DNA removal

Ribozol (VMR) was used to extract total RNAs from both the total synovial fluid (both cell and extracellular matrix) and chondrocytes, followed by the digestion of genomic DNA with DNase I (Invitrogen) at 37°C for 2 h. Electrophoresis was performed using urea-PAGE gels (5%) to analyze RNA integrity. RNA purity was analyzed by measuring the OD260/280 ratios.

### 2.5 RT-qPCRs

Only RNA samples with an OD260/280 ratio close to 2.0 were used as templates to prepare cDNA samples through reverse transcriptions (RTs). RTs were performed using a PrimeScript RT-PCR Kit (Takara Bio) with 1,000 ng of RNA from each RNA sample as a template. RT thermal conditions were 5 min at 25°C, 20 min at 55°C, and 10 min at 80°C. The expression of Circ_0038467, BAX, and BCL2, qPCR was determined using SYBR Green Master Mix (Bio-Rad). GAPDH was used as an internal control. To determine the expression of the miR-203 precursor, the same method was used to perform RTs and qPCRs with sequence-specific primers.

To determine the expression of mature miR-203, All-in-One™ miRNA qRT-PCR Detection Kit* (GeneCopoeia) was used to perform poly (A) addition, followed by using poly (T) as a reverse primer to carry out RT-qPCR. U6 was used as the internal control.

Primer sequences were the following: Circ_0038467: 5′‐TCCCAGCTGACCTAAAGTCAAT‐3′ (forward) and 5′‐TGGTGACATTGAGCAGGAAC‐3′ (reverse), BCL2: 5′‐CTTCCAGGAACCTCTGTGATG‐3′ (forward) and 5′‐AATGCCGCCATCGCTTACACC-3′ (reverse), Bax2: 5′‐TGAGCGAGTGTCTCAAGCG‐3′ (forward) and 5′‐CCCCAGTTGAAGTTGCCGT‐3′ (reverse), GAPDH: 5′‐GTCAGCCGCATCTTCTTTTG‐3′ (forward) and 5′‐GCGCCCAATACGACCAAATC‐3′ (reverse); miR-203 precursor: 5′‐TGTGTTGGGGACTCGCGCGCGT‐3′ (forward) and 5′‐TCGCTGTCGCCGCGCGCC‐3′ (reverse); mature miR-203:5′‐AGTGGTTCTTAACAGTTCAAC‐3′ (forward) and poly (T). U6 primers were obtained from the kit. PCR thermal conditions were as follows: 1 min at 95°C, followed by 10 s at 95°C, and 40 s at 59°C for a total of 40 cycles. The *C*_t_ values of target genes were normalized to internal controls using the 2^−ΔΔCt^ method. The sample with the highest Δ*C*t value was set to value “1,” and all other samples were normalized to this sample.

### 2.6 Apoptosis assay

Apoptosis of chondrocytes was analyzed at 48 h post-transfection. In brief, chondrocytes were washed with PBS and cultivated in a medium supplemented with 10 ng/ml LPS for 48 h. Three replicate wells were set for each experiment. After that, chondrocytes were washed with pre-cold PBS and stained with Annexin-V FITC and then propidium iodide (PI) for 12 min in the dark. Finally, cell apoptosis was analyzed using a FACSCalibur instrument. FCS Express Flow Cytometry Software (De Novo Software) was used to process flow cytometry data.

### 2.7 Subcellular fractionation analysis

Nucleus and cytoplasm fractions were prepared using a Cell Fractionation Kit purchased from Abcam (ab109719). All operations were performed following the manufacturer’s instructions. Both fractions were used to isolate RNA samples, which were subjected to RT-PCRs to determine the expression of Circ_0038467. GAPDH was used as a cytoplasm marker.

### 2.8 Biotin RNA–RNA pull-down

Biotin-labeled NC RNA (Bio-NC) and miR-203 precursor (Bio-miR-203-pre) were prepared and transfected into cells. Cell lysates were prepared 48 h later and incubated with Dynabeads to pulldown RNA complex. RT-qPCR was performed to measure the levels of Circ_0038467 accumulation.

### 2.9 Statistical analysis

Data were presented as the mean ± standard deviation or count (percentage). An unpaired *t*-test was used to compare OA and the control group. Data from multiple independent groups were analyzed by ANOVA (one-way or two-way depending on the number of independent variables) Tukey’s test. Correlation analysis was performed using Pearson’s correlation coefficient. *p* < 0.05 was considered statistically significant.

## 3 Results

### 3.1 Circ_0038467 was upregulated in OA and was downregulated after the treatment

**Figure 1:**
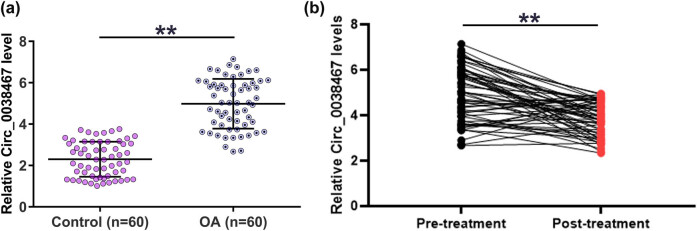
Circ_0038467 was upregulated in OA and was downregulated after the treatment. Circ_0038467 expression was studied by RT-qPCR. An unpaired *t*-test was used to compare two groups (a). ** *p* < 0.01. All patients were treated with NSAIDs. Circ_0038467 expression in the synovial fluid samples from OA patients was also determined by RT-qPCR 1 month after treatment. Paired *t-*test was applied to compare pre-treatment and post-treatment levels of Circ_0038467 (b). RT-qPCR was performed in three technical replicates and average values were presented and compared.

Altered gene expression reflects the function. To this end, RT-qPCR was performed to explore the differential expression of Circ_0038467 in OA. The results showed that Circ_0038467 was upregulated in OA compared to that in the control group (2.12-fold, Figure 1a, *p* < 0.01). All patients were treated with NSAIDs. At 1 month after treatment, symptoms were improved based on the degree of pain of OA in all cases. Circ_0038467 expression in synovial fluid samples from OA patients was also determined by RT-qPCR. It showed that the expression levels of Circ_0038467 were decreased after the treatment (post-treatment) compared to the pre-treatment level (Figure 1b, *p* < 0.01, paired *t*-test). Therefore, the upregulation of Circ_0038467 may participate in OA. Measuring the expression of Circ_0038467 may reflect the treatment efficiency of OA.

### 3.2 Mature miR-203 and miR-203 precursor were upregulated in OA but only mature miR-203 was positively correlated with Circ_0038467

**Figure 2:**
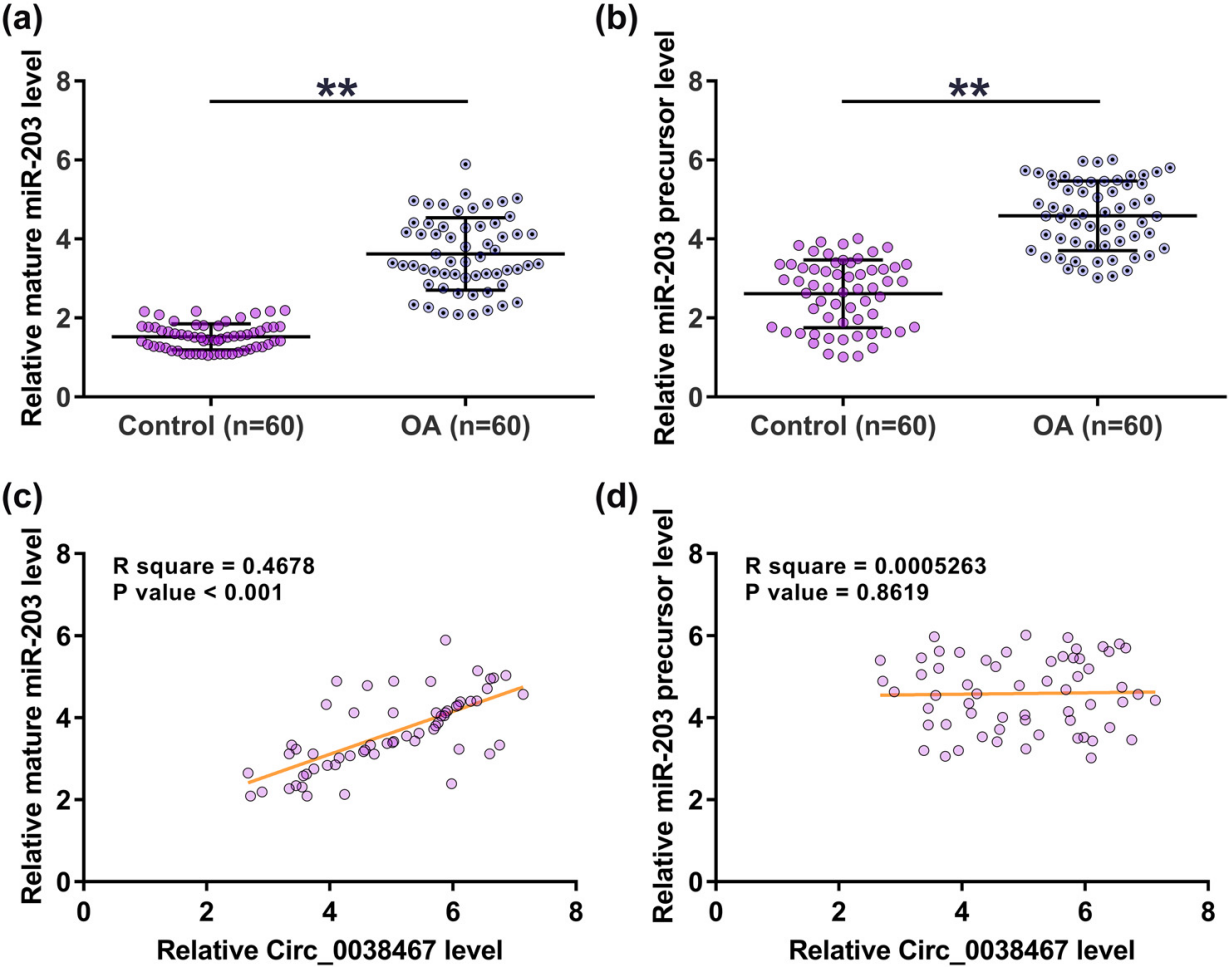
Mature miR-203 and miR-203 precursor were upregulated in OA but only mature miR-203 was positively correlated with Circ_0038467. Mature miR-203 and the miR-203 precursor expressions were studied by RT-qPCR. An unpaired *t*-test was used to compare the expression levels of mature miR-203 (a) and miR-203 precursor (b) between two groups. RT-qPCR was performed in three technical replicates, and average values were presented and compared. ** *p* < 0.01. Pearson’s correlation coefficient analysis was performed to analyze the correlations between Circ_0038467 and mature miR-203 (c) or miR-203 precursor (d) across the synovial fluid samples from OA patients.

RT-qPCR was performed to explore the differential expression of mature and precursor miR-203 in OA. It showed that mature miR-203 (Figure 2a) and miR-203 precursor (Figure 2b) were both upregulated in OA compared to that in the control group (2.23- and 1.97-fold, respectively, *p* < 0.01). Pearson’s correlation coefficient analysis showed that the expression of Circ_0038467 was positively correlated with mature miR-203 (Figure 2c) but not miR-203 precursor (Figure 2d) across synovial fluid samples from OA patients. The abundance of Circ_0038467 and mature miR-203 in synovial fluid samples from the 12 OA patients was analyzed by deep sequencing, which also revealed the close correlation between them across these 12 OA samples (Figure S1). Therefore, it is reasonable to hypothesize that Circ_0038467 may participate in the maturation of miR-203.

### 3.3 Overexpression of Circ_0038467 upregulated the expression of mature miR-203 in chondrocytes

**Figure 3:**
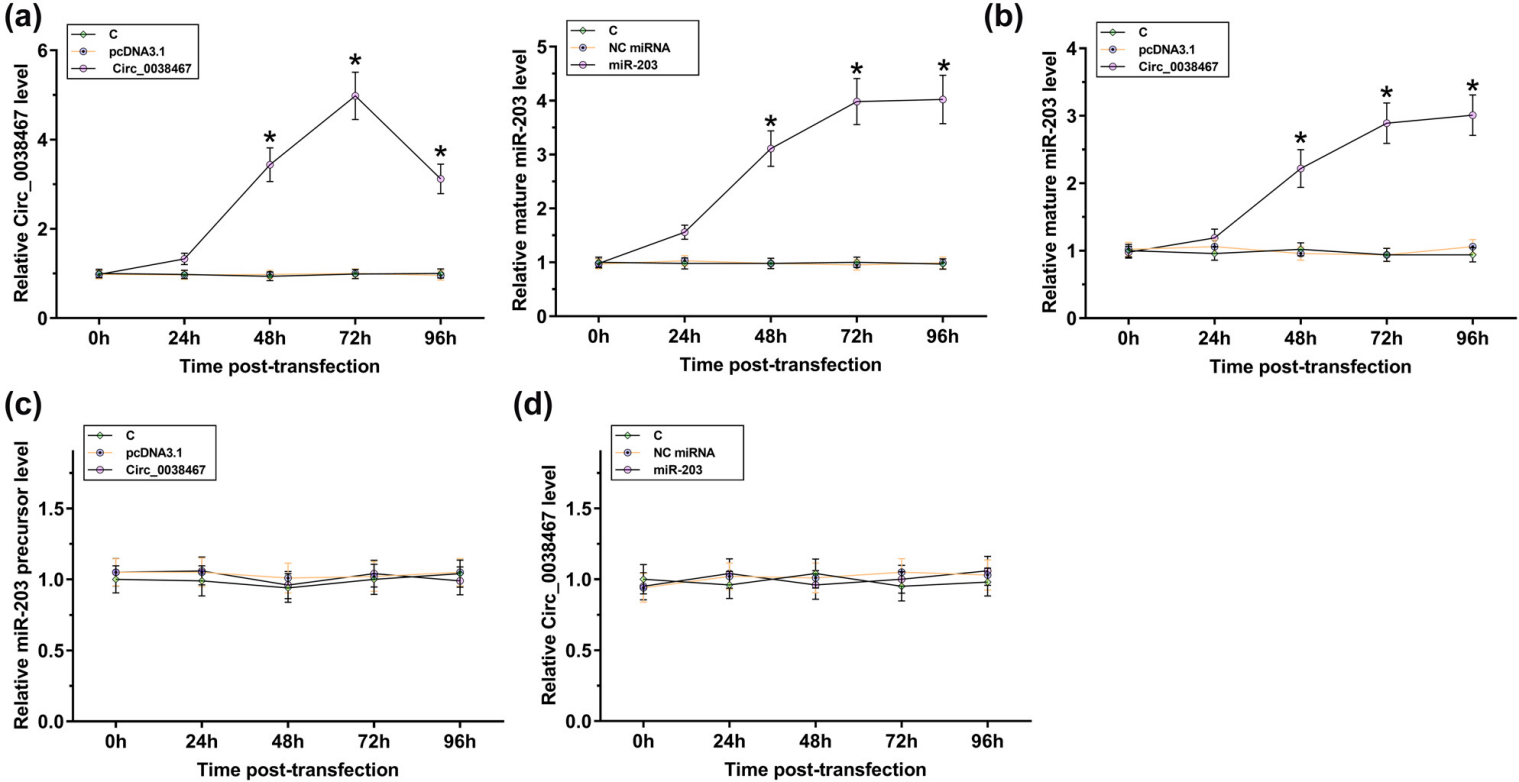
Overexpression of Circ_0038467 upregulated the expression of mature miR-203 in chondrocytes. The Circ_0038467 expression vector or miR-203 mimic was transfected into chondrocytes, followed by the confirmation of overexpression of Circ_0038467 (a) and miR-203 (b) every 24 h until 96 h. The effects of overexpression of Circ_003846 on the expression of mature miR-203 (b) and miR-203 precursor (c) and the effects of overexpression of miR-203 on Circ_0038467 (d) were analyzed by RT-qPCR at each time point. Three biological replicates were performed. Mean ± SD values were presented and compared by ANOVA Tukey’s test. *p < 0.05.

The close correlation between Circ_0038467 and mature miR-203 suggests the potential interaction between them. To this end, cells were transfected with the Circ_0038467 expression vector or miR-203 mimic, and the overexpression of Circ_0038467 and miR-203 was confirmed by performing RT-qPCR every 24 h until 96 h. It was observed that Circ_0038467 and miR-203 were successfully overexpressed between 48 and 96 h (more than 3-fold at 96 h, Figure 3a, *p* < 0.05). Moreover, overexpression of Circ_0038467 increased the expression levels of mature miR-203 from 48 to 96 h (more than 2.5-fold at 96 h, Figure 3b, *p* < 0.05) but did not affect the expression of miR-203 precursor at each time point (Figure 3c). Moreover, overexpression of miR-203 did not affect the expression of Circ_0038467 at each time point (Figure 3d). Therefore, Circ_0038467 promoted the maturation of miR-203 in chondrocytes.

### 3.4 Overexpression of Circ_0038467 increased LPS-induced apoptosis of chondrocytes through miR-203

**Figure 4:**
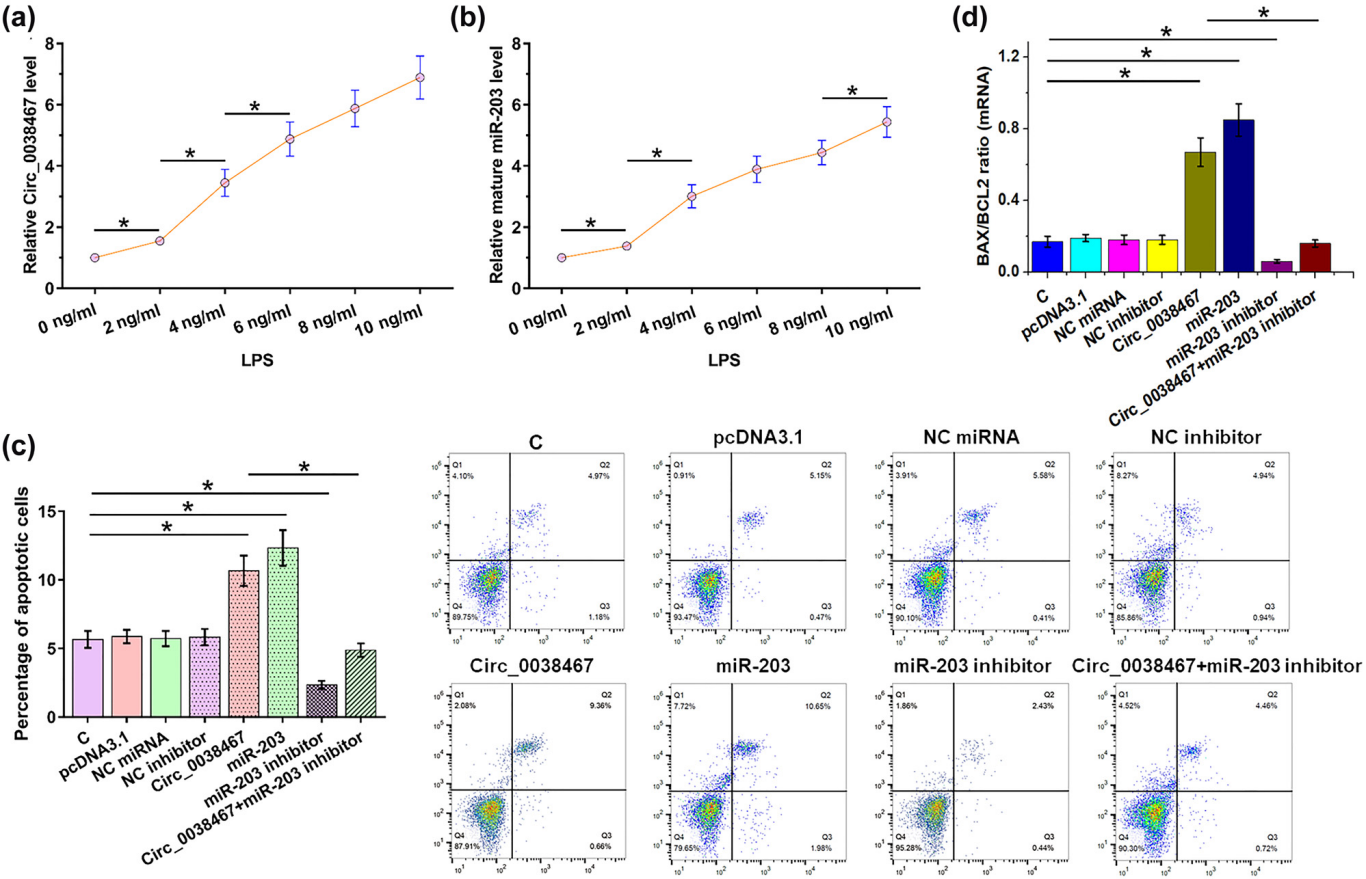
Overexpression of Circ_0038467 increased LPS-induced apoptosis of chondrocytes by miR-203. To study the effects of LPS treatment on the expression Circ_0038467 and miR-203, chondrocytes were cultivated in a medium containing 0, 2, 4, 8, and 10 ng/ml LPS for 48 h, followed by the determination of the expression of Circ_0038467 (a) and miR-203 (b) by RT-qPCR. The role of Circ_0038467 and miR-203 in regulating the apoptosis of chondrocytes was analyzed by the cell apoptosis assay using cells with transfection (c). In each group included in the cell apoptosis assay, the expression of BAX and BCL2 was analyzed using RT-qPCR at the mRNA level. The BAX/BCL2 ratio, which reflects cell apoptosis, was calculated (d). Three biological replicates were performed. Mean ± SD values were presented and compared by ANOVA Tukey’s test. *p < 0.05.

To study the effect of LPS treatment on the expression of Circ_0038467 and miR-203, chondrocytes were cultivated in a medium with LPS for 48 h, followed by detection of the expression of Circ_0038467 (Figure 4a) and miR-203 (Figure 4b) by RT-qPCR. It was observed that LPS increased the expression levels of Circ_0038467 and miR-203 in a dose-dependent manner (0.7- to 7.1-fold and 0.6- to 5.3-fold, respectively, *p* < 0.05). Analysis of the apoptosis of chondrocytes induced by LPS showed the overexpression of Circ_0038467 and miR-203, which was achieved by transfecting the Circ_0038467 expression vector or miR-203 mimic into cells, increased cell apoptosis. In addition, the miR-203 inhibitor reversed the effects of overexpression of Circ_0038467 on cell apoptosis (Figure 4c, *p* < 0.05). Expression of BAX and BCL2 was analyzed using RT-qPCR at the mRNA level. The BAX/BCL2 ratio, which reflects cell apoptosis, was calculated. Circ_0038467 and miR-203 increased the BAX/BCL2 ratio. In addition, the miR-203 inhibitor reversed the effects of overexpression of Circ_0038467 on the BAX/BCL2 ratio (Figure 4d, *p* < 0.05). Therefore, Circ_0038467 may promote the LPS-induced apoptosis of chondrocytes through miR-203. Representative images of the cell apoptosis assay are listed in Supplemental File 1.

### 3.5 Circ_0038467 in the nucleus may sponge the miR-203 precursor

**Figure 5:**
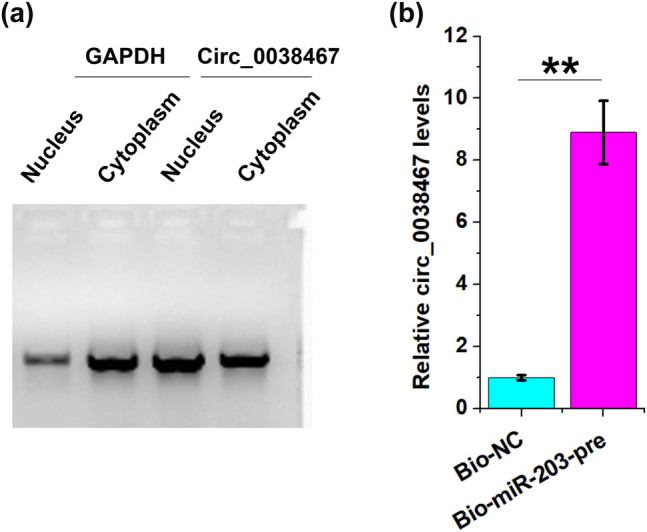
Circ_0038467 in the nucleus may sponge the miR-203 precursor. The subcellular location of Circ_0038467 was analyzed by performing a subcellular fractionation assay (a). The direct interaction between Circ_0038467 and miR-218 precursor (miR-218-pre) was analyzed by the RNA pulldown assay (b). **p < 0.01.

The subcellular location of Circ_0038467 was analyzed by performing a subcellular fractionation assay. It was observed that Circ_0038467 can be detected in both the cytoplasm and nucleus (Figure 5a). The direct interaction between Circ_0038467 and the miR-218 precursor (miR-218-pre) was analyzed by RNA-pulldown assay. It was observed that Circ_0038467 and miR-218-pre can directly interact with each other (Figure 5b, *p* < 0.05). Therefore, Circ_0038467 may carry the miR-218 precursor transporting from the nucleus to cytoplasm, thereby promoting its maturation.

## 4 Discussion

In this study, the differential expression of Circ_0038467 and miR-203 in OA, as well as the potential interactions between them were explored. We observed that both Circ_0038467 and miR-203 were upregulated in OA. Interestingly, Circ_0038467 may promote the maturation of miR-203 to increase the apoptosis of chondrocytes induced by LPS.

Liu et al. reported that Circ_0038467 was upregulated in human bronchial epithelial cells by LPS treatment, and knockdown of Circ_0038467 protected the cells from LPS-induced injury [13], suggesting that Circ_0038467 may promote LPS-induced cell injury. Chondrocytes are the only mature cells found in the cartilage [14]. The main function of chondrocytes is to maintain and sustain the cartilage by secreting extracellular matrix [14]. During OA, apoptosis of chondrocytes will be increased, leading to the degradation of the cartilage matrix [14]. LPS is known to induce the degradation of the cartilage matrix. However, the mechanism has not been fully elucidated. The present study showed that LPS treatment increased the expression levels of Circ_0038467 in chondrocytes in a dose-dependent manner. Moreover, overexpression of Circ_0038467 increased the apoptosis of chondrocytes under LPS treatment. In addition, Circ_0038467 was also upregulated in OA patients compared to that in healthy controls. Therefore, LPS-induced Circ_0038467 may promote OA by increasing the apoptosis of chondrocytes. It also suggested that Circ_0038467 may serve as a potential target to treat OA.

Knockdown of miR-203 can target MCL-1 to reduce LPS-induced injury in chondrocytes [15], suggesting the involvement of miR-203 in OA. In this study, miR-203 was reported to be upregulated in OA at both precursor and mature miRNA levels. Moreover, the expression of miR-203 can also be induced by LPS, and overexpression of miR-203 increased the apoptosis of chondrocytes under LPS treatment. Therefore, miR-203 may also promote OA progression by increasing LPS-induced apoptosis of chondrocytes.

Interestingly, overexpression of Circ_0038467 was found to increase the expression levels of mature miR-203 but not that of miR-203 precursor in chondrocytes. Therefore, Circ_0038467 may suppress the maturation of miR-203. The possible reasons why Circ_0038467 was not closely correlated with the miR-203 precursor may include altered transcription and/or RNA degradation. Considering the fact that the maturation of miRNAs requires the movement of the miRNA precursor from the nucleus to the cytoplasm, Circ_0038467 may promote the transportation of the miR-203 precursor to increase its maturation. This hypothesis was also confirmed in this study. However, the *in vivo* interaction between Circ_0038467 and miR-203 is unclear. Animal model experiments are needed.

## 5 Conclusion

In conclusion, Circ_0038467 and miR-203 are upregulated in OA. In addition, Circ_0038467 may promote the maturation of miR-203 to increase the apoptosis of chondrocytes induced by LPS, thereby promoting the progression of OA. This study is the first to report the participation of Circ_0038467 and miR-203 in OA. In addition, this research for the first time showed the regulation of miR-203 maturation by CircRNA. Future studies may focus on the clinical application of the Circ_0038467/miR-203 axis for the diagnosis and treatment of OA.

## Supplementary Material

Supplementary Figure
