# Supplementary material for: Circular RNA Circ_0038467 promotes the maturation of miRNA-203 to increase lipopolysaccharide-induced apoptosis of chondrocytes
Source: Open Med (Wars). 2023 Jun 5;18(1):20220557. doi: 10.1515/med-2022-0557 (PMC10251158; doi:10.1515/med-2022-0557)
Supplement: Supplementary Figure [file med-2022-0557-sm.pdf]

Supplementary material

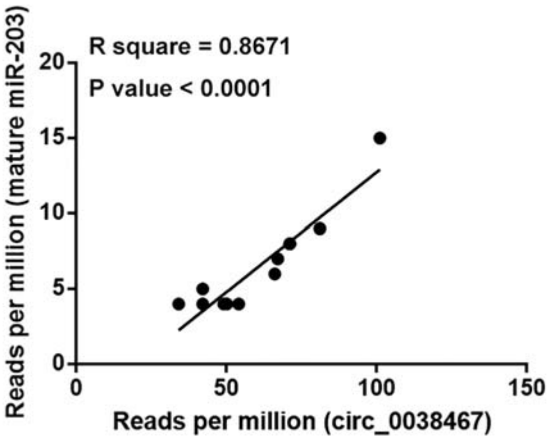

Figure S1: Correlation analysis between circ_0038467 and mature miR-203 across 12 OA samples using deep sequencing data.

Supplemental File 1. Representative images of cell apoptosis assay

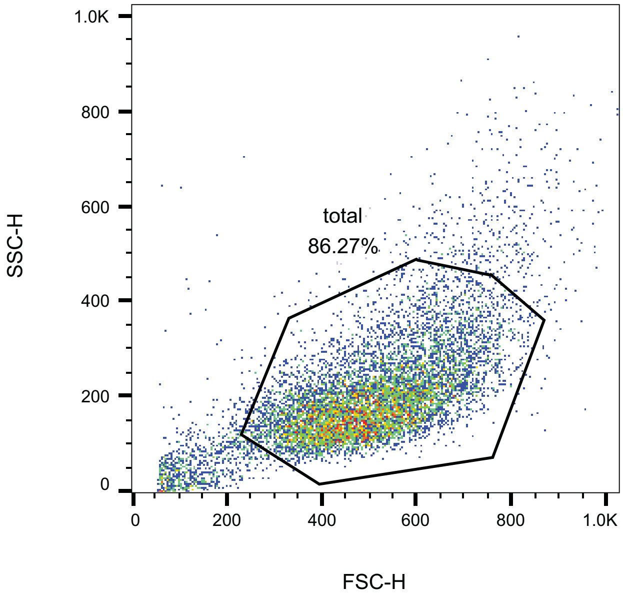

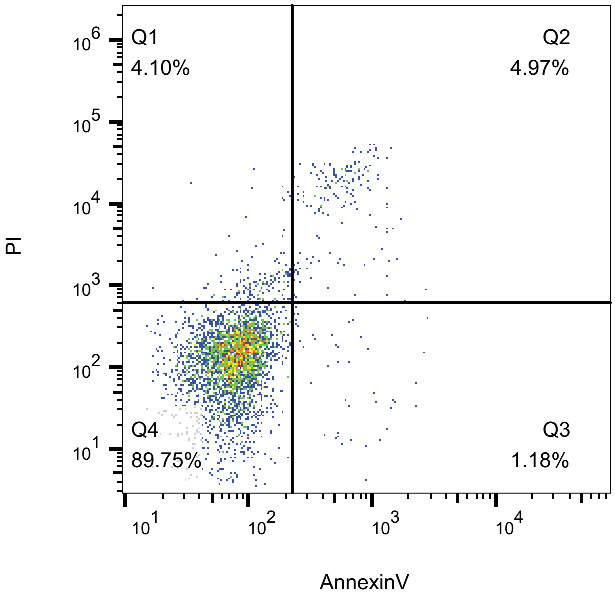

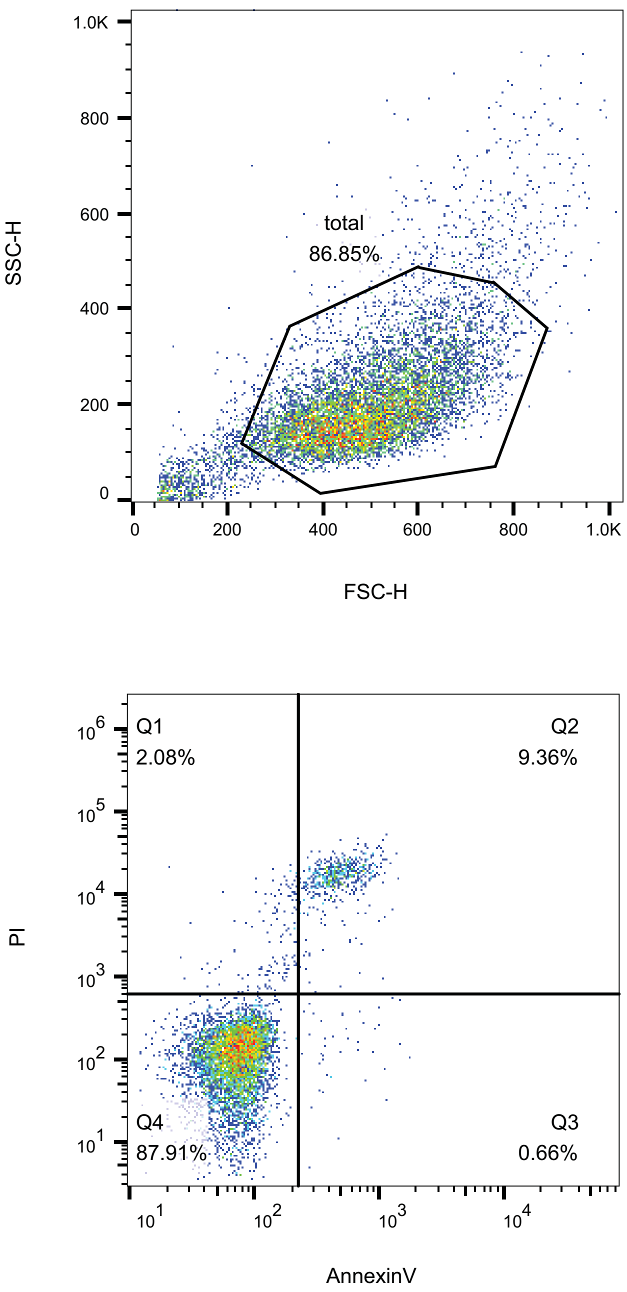
SSC-H
FSC-H
total
86.85%
0
200
400
600
800
1.0K
PI
AnnexinV
Q1
2.08%
Q2
9.36%
Q4
87.91%
Q3
0.66%

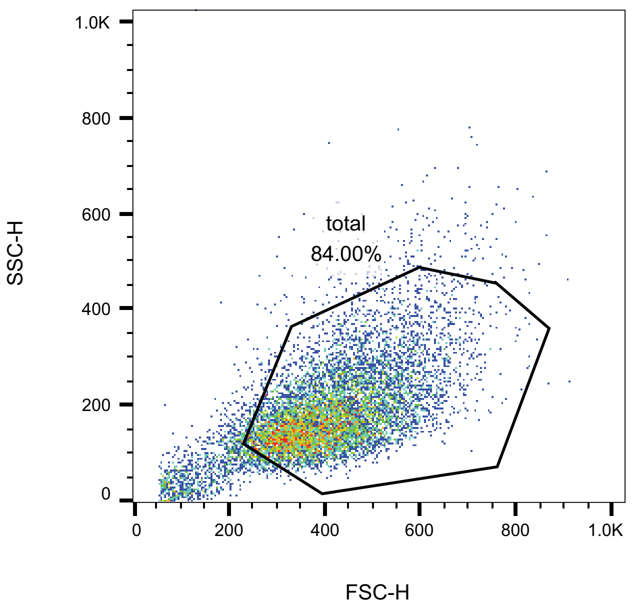
1.0K
800
600
400
200
0
SSC-H
total
84.00%
0
200
400
600
800
1.0K
FSC-H

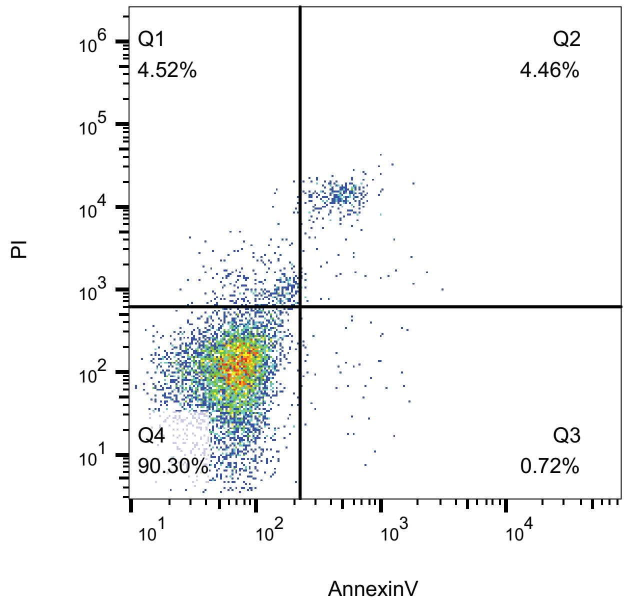
Q1
4.52%
Q2
4.46%
Q4
90.30%
Q3
0.72%
PI
AnnexinV

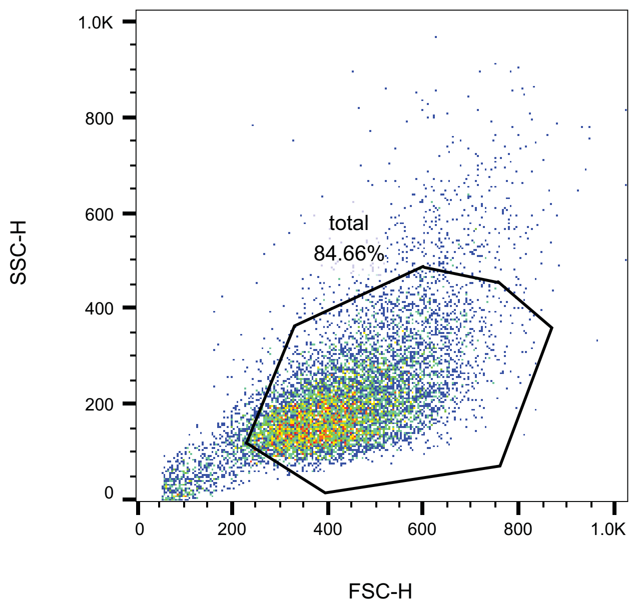
1.0K
800
600
400
200
0
SSC-H
total
84.66%
0
200
400
600
800
1.0K
FSC-H

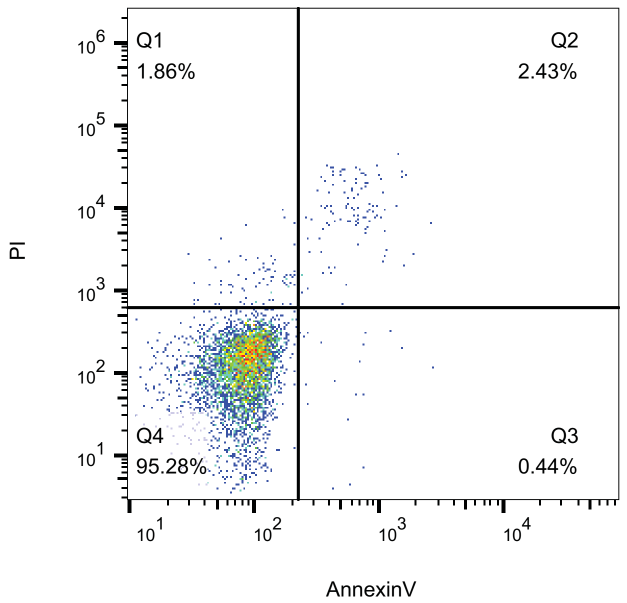
Q1
1.86%
Q2
2.43%
Q3
0.44%
Q4
95.28%
PI
AnnexinV

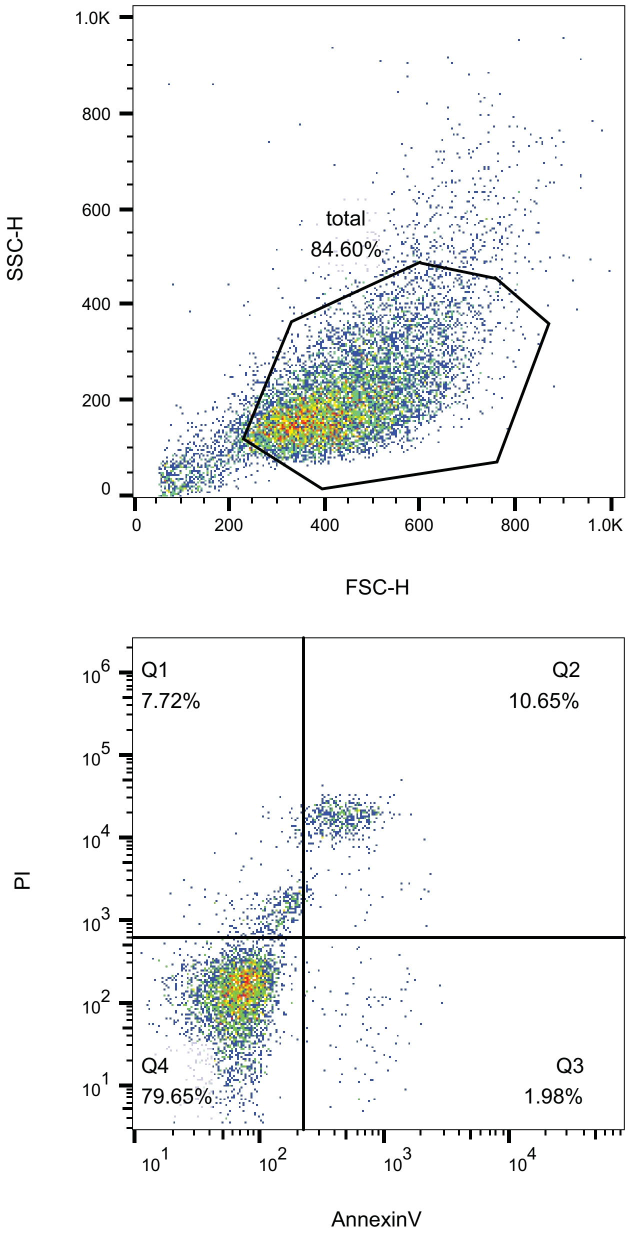
1.0K
800
600
400
200
0
SSC-H
total
84.60%
0
200
400
600
800
1.0K
FSC-H
10^6
10^5
10^4
10^3
10^2
10^1
PI
Q1
7.72%
Q2
10.65%
Q4
79.65%
Q3
1.98%
10^1
10^2
10^3
10^4
AnnexinV

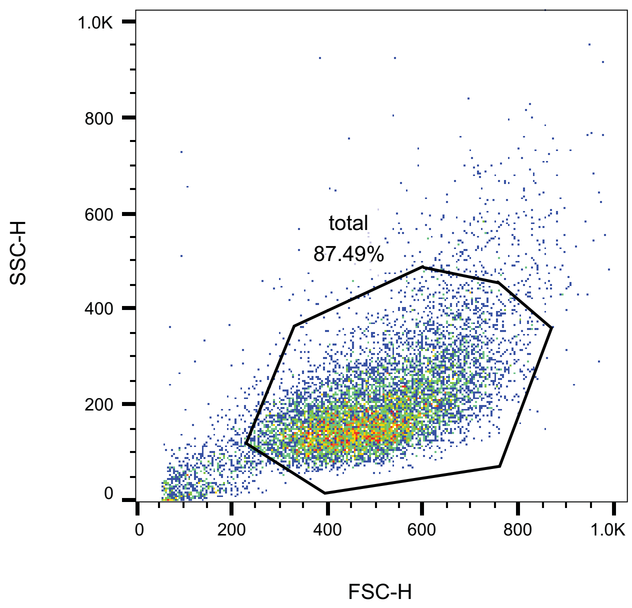
SSC-H
FSC-H
total
87.49%
0
200
400
600
800
1.0K

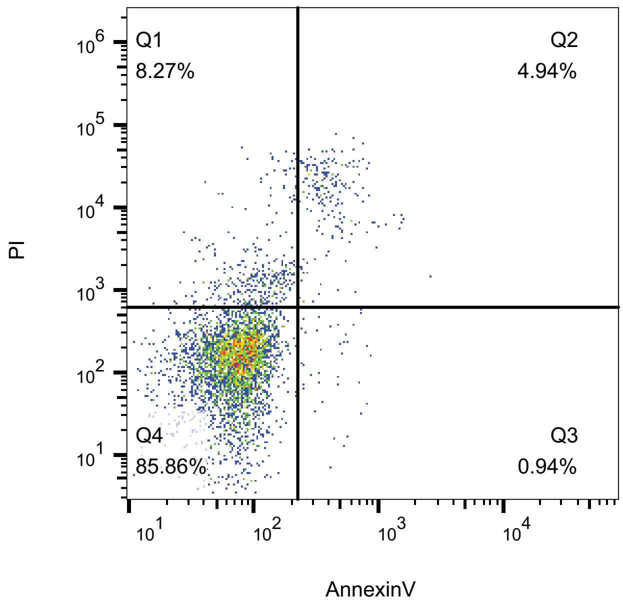
PI
AnnexinV
Q1
8.27%
Q2
4.94%
Q3
0.94%
Q4
85.86%

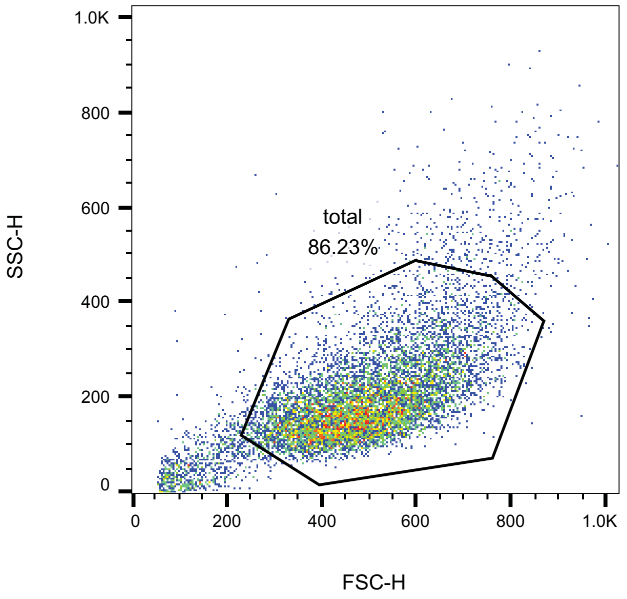
total
86.23%
1.0K
800
600
400
200
0
SSC-H
0
200
400
600
800
1.0K
FSC-H

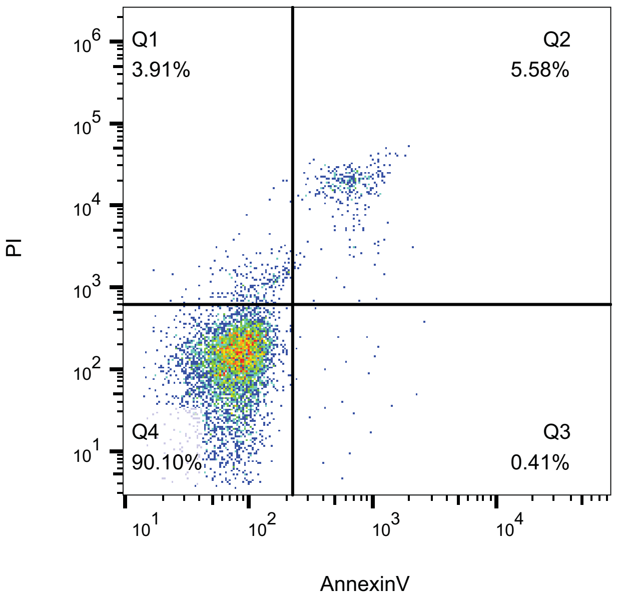
Q1
3.91%
Q2
5.58%
Q4
90.10%
Q3
0.41%
PI
AnnexinV

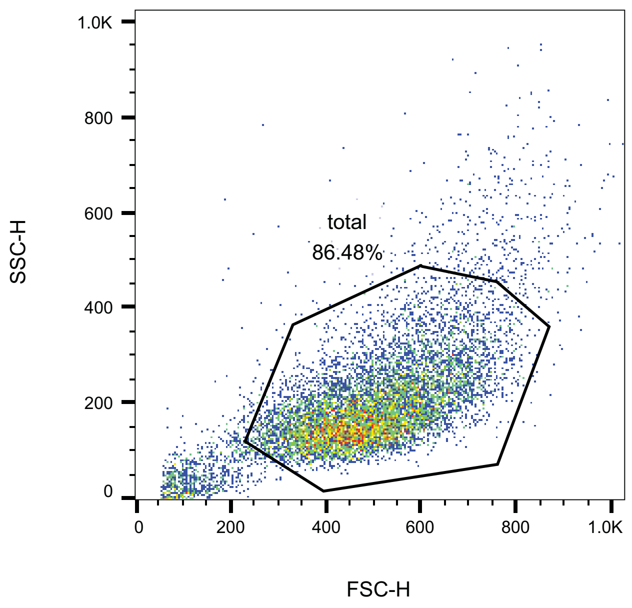
1.0K
800
600
400
200
0
SSC-H
total
86.48%
0
200
400
600
800
1.0K
FSC-H

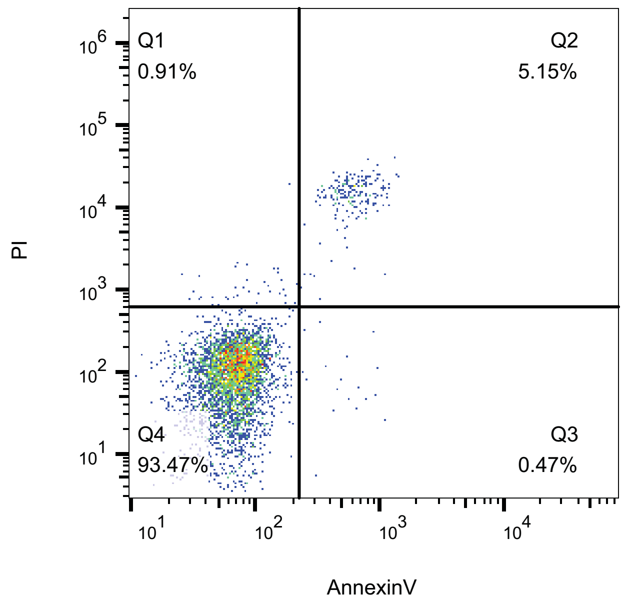
Q1
0.91%
Q2
5.15%
Q4
93.47%
Q3
0.47%
PI
AnnexinV
